# Fluids of the Future

**DOI:** 10.3389/fvets.2020.623227

**Published:** 2021-01-21

**Authors:** Thomas H. Edwards, Guillaume L. Hoareau

**Affiliations:** ^1^US Army Institute of Surgical Research, San Antonio, TX, United States; ^2^Emergency Medicine, School of Medicine, University of Utah, Salt Lake City, UT, United States

**Keywords:** blood product development, hemorrhage, innovation, resuscitation, sepsis, shock, traumatic brain injury

## Abstract

Fluids are a vital tool in the armament of acute care clinicians in both civilian and military resuscitation. We now better understand complications from inappropriate resuscitation with currently available fluids; however, fluid resuscitation undeniably remains a life-saving intervention. Military research has driven the most significant advances in the field of fluid resuscitation and is currently leading the search for the fluids of the future. The veterinary community, much like our civilian human counterparts, should expect the fluid of the future to be the fruit of military research. The fluids of the future not only are expected to improve patient outcomes but also be field expedient. Those fluids should be compatible with military environments or natural disaster environments. For decades, military personnel and disaster responders have faced the peculiar demands of austere environments, prolonged field care, and delayed evacuation. Large scale natural disasters present field limitations often similar to those encountered in the battlefield. The fluids of the future should, therefore, have a long shelf-life, a small footprint, and be resistant to large temperature swings, for instance. Traumatic brain injury and hemorrhagic shock are the leading causes of preventable death for military casualties and a significant burden in civilian populations. The military and civilian health systems are focusing efforts on field-expedient fluids that will be specifically relevant for the management of those conditions. Fluids are expected to be compatible with blood products, increase oxygen-carrying capabilities, promote hemostasis, and be easy to administer in the prehospital setting, to match the broad spectrum of current acute care challenges, such as sepsis and severe systemic inflammation. This article will review historical military and civilian contributions to current resuscitation strategies, describe the expectations for the fluids of the future, and describe select ongoing research efforts with a review of current animal data.

## The Drive for the Fluids of the Future

### Military Research and Needs

Military research has driven much of the resuscitation advances over the years. Large scale conflicts and field-specific demands have outlined the need to address common conditions. Hemorrhagic shock, traumatic brain injury, burns, and sepsis are responsible for the vast majority of combat casualties from the most recent conflicts in the Middle East (1–3). Hemorrhage remains by far the leading cause of preventable deaths (1). Those observations lead the military trauma community to develop field-expedient solutions relevant to operative constraints.

### Civilian Resuscitation

While resuscitation differs drastically between a warzone and a civilian setting, there is still a need for novel resuscitation fluids in non-combat scenarios. Patient care at the point-of-injury and during transport to definitive care can also be challenging during non-military operations. This is illustrated by massive casualties scenarios where blood supplies and resuscitation teams are rapidly exhausted. Resuscitation resources may be overwhelmed and exhausted following natural (hurricanes, flooding, etc.), or human-made disasters (terrorist attacks). Resuscitation in underserved areas, such as rural communities or Native American reservations, presents similar challenges. Care-providers practicing in those environments will likely benefit from field-expedient novel fluids. Most deaths occur within the first 6 h following trauma (4), which emphasizes the importance of adequate resuscitation tools near the point-of-injury.

### Veterinary Resuscitation

Military working dogs are exposed to significant hazards during deployment and suffer similar injuries as their human counterparts. Hemorrhagic shock and sepsis are also concerning in those patients. There is limited research explicitly pertaining to canine resuscitation in austere environments and prolonged field care scenarios.

Civilian veterinary populations will also benefit from advances toward fluids of the future. Law enforcement and search and rescue dogs may present similar injury patterns as military working dogs (5). They often operate in austere environments with limited resources and may suffer similar injuries (gun, stab wounds, burns, impalement, etc.). Aside from potential medical benefits over current products, fluids of the future are also likely to be used in smaller clinics that cannot carry products with demanding storage requirements or a short shelf life.

## The Ideal Fluid

Shock is defined by the American College of Surgeons as the inadequate perfusion of tissue, such that the oxygen and blood volume delivery fail to meet the cellular metabolic and oxygen consumption needs. This definition does not however account for cytopathic shock, whereby cells may not be able to produce ATP despite normal oxygen delivery (6–8). Therefore, the treatment of shock focuses on restoring tissue oxygen delivery by improving blood volume and flow in both the macro and microcirculation. Sustaining arterial content of oxygen is a cornerstone of resuscitation as well. However, despite the normalization of markers of tissue oxygen delivery (blood pressure, hemoglobin concentration, arterial partial pressure in oxygen, etc.), patients may suffer ongoing organ injury due to microcirculation and mitochondrial disorders. The ideal fluids should provide a solution to the various components of the physiopathology of shock. While the quest for the ideal fluid is still ongoing, its characteristic can be outlined in Table 1.

**Table 1:** Desirable properties of an ideal resuscitation fluid.

**Example of characteristics for the ideal resuscitation fluid**
No storage lesion for prolonged periods at ambient temperatures up to 130°F (54°C)
Ready or easily prepared for use
Universally compatible
Support oxygen-carrying capacity
Mitigates or negates post-shock, post-resuscitation syndromes
Compatible with blood products
No impairment of coagulation capability
Minimal or mitigating effect on edema
Improves microcirculation
Enhance cellular resuscitation
Support mitochondrial function
Components fully eliminated from the body
Suitable for administration by a wide range of medical personnel
No behavioral or physical post-resuscitation impairment
Maintain or restore normal species dependent values for:Electrolytes concentrations Osmolarity Tonicity Colloid osmotic pressure Viscosity Acid-Base homeostasis Caloric requirements

## Novel Crystalloids

Crystalloids, both hyper- and isotonic, have been extensively studied for the management of various types of fluid-responsive shocks. Advances in knowledge have also underlined their limitations, such as hemodilution, blood acidification, hypothermia, coagulopathies, etc. (9). Those side effects serve as a canvas for research and development efforts.

Recently a crystalloid that supports nitric oxide synthesis (by containing nitrate and nitrite) and free-radical scavenging (by containing magnesium and transition metals) was similar to whole blood at improving hemodynamic markers of hypoperfusion (mean arterial pressure, serum lactate, central venous oxygen saturation) in pigs subjected to hemorrhagic shock (10). This product showed similar histological markers of tissue and glycocalyx injury compared to whole blood. Such effort seeks to address microcirculatory dysfunction observed during states of shock, while utilizing a non-hemoglobin-based resuscitation tool.

Another approach has been to add polymers of polyethylene glycol (PEG) to exert osmotic and hydrophilic actions in the microcirculation. Much remains to be done regarding the coagulation effects of the product (11); pigs subjected to hemorrhagic shock and resuscitated with the investigational product had reduced maximal amplitudes when compared to control animals. The solution still showed promising results in pre-clinical studies comparing it to whole blood or starches (12). Survival rate at 24 h were significantly higher when pigs were resuscitated from hemorrhagic shock with the PEG product, when compared to whole blood and hextend (100, 17, and 0%, respectively). The PEG solution also improved mean arterial pressure and capillary perfusion when compared to the other products.

Hypertonic saline is a product that has been heavily studied in brain injury resuscitation. Hypertonic saline (3.5–7%) has been enriched with adenosine, lidocaine, and magnesium. The use of this formula, named ALM therapy, also led to promising results in pre-clinical rodent and swine models. The solution extends survival, reduces ongoing hemorrhage, modulate coagulopathy, and prevents immunodeficiency (13). Encouraging benefits have been observed in rodent models of traumatic brain injury with increased survival, cardiac function, and cortical blood flow (14). Conversely, in pigs receiving lipopolysaccharide, ALM therapy led to a reversible hypotensive and hypometabolic state, with reduced markers of inflammation (15). This exemplifies that a given product is likely to be beneficial in specific shock scenarios. There is a paucity of data explaining the actual mechanism of action of the drug; modulation of damage-associated molecular pathways and reduction in the inflammasome (Multiprotein complex that activates the pro-inflammatory cytokines interleukin 1b and 18) activity might contribute to clinical effects (16).

## Novel Blood Products/Blood Product Replacement

Current evidence strongly supports using blood products over crystalloids or synthetic colloids to treat human patients with hemorrhagic shock in both civilian and military patients. There is ample evidence showing the deleterious effects of over resuscitation with isotonic crystalloids associated with edema, cellular, metabolic, and immune dysfunction (17). Analysis of military casualties has shown that the use of blood in the treatment of combat casualties has saved lives. In a recent report, investigators estimated that 873 additional service members would have died in the wars in Iraq and Afghanistan had they not received blood products for their traumatic injuries (18). The clinical benefits of blood conflict with their use: they come in limited supply or might be unavailable altogether in combat, are complicated to store, have a limited shelf life, and can be associated with bacterial contamination (19, 20). The development of field-expedient blood products or substitutes is a primary focus for the military community. Synthetic products allow control of the supply chain while avoiding the risk of transferring disease from blood donor to the recipient.

### Red Blood Cells Replacement Products

The development of hemoglobin-based oxygen carriers (HBOC) has been a dynamic field for decades. Numerous products showed promises in animal models but unfortunately were not successful in human clinical trials. A single meta-analysis, which has since been criticized (21–23), cast serious doubt on the clinical benefits of HBOC while outlining a high incidence of complications (24). Therefore, there is currently no FDA-approved HBOC in clinical use in human or veterinary medicine. Lack of clinical benefit has been attributed to nitric oxide-scavenging properties of hemoglobin leading to hypertension, oxygen oversupply, heme-mediated oxidative stress (25–27). Another limitation of earlier HBOC was the lack of dynamic oxygen affinity. In health, the hemoglobin saturation curve is sigmoid due to its allosteric effector 2,3-Diphosphoglycerate (2,3-DPG) and collaborative binding of oxygen (i.e., cooperativity: binding of the first oxygen molecule facilitates the binding of the next one, etc.). First-generation HBOC did not present such properties (28). Common side effects reported after using a wide range of HBOC products include gastrointestinal symptoms, pancreatic and liver enzyme elevation, myocardial infarction, arrhythmias, renal injury, and death (29).

While clinical complications were outlined, this meta-analysis also provided the industry with guidance to improve future products. Refinement involved chemical alterations to prolong half-lives such as polymerization or linking to non-protein molecules such as PEG or polyoxyethylene. HBOCs can be administered with antioxidant molecules, such as ascorbic acid, to prevent oxidative stress (30). Cross-linking with antioxidant enzymes, such as superoxide dismutase or catalase, has also been investigated in a rat model of severe cerebral ischemia-reperfusion showing improved perfusion and injury mitigation (31).

A PEGylated carboxyhemoglobin compound (Sanguinate™) is in clinical development. PEGylation prolongs circulating half-life by interfering with cell surface receptors involved in removal from circulation, prevents extravasation by increasing molecular weight, and reduces immunogenicity. Carboxylation prevents the formation of methemoglobin during storage. This molecule also releases carbon monoxide; while at high concentration, carbon monoxide is toxic, at lower concentrations, it protects cells from ischemia by mitigating inflammation, oxidative stress, and apoptosis (32). In rats subjected to hemorrhagic shock, PEGylated carboxyhemoglobin reduced evidence of renal injury (tubular histologic damage as well as neutrophil gelatinase-associated lipocalin and tumor necrosis factor alpha immunohistochemistry) despite not improving renal tissue oxygenation (33). In another study, PEGylated carboxyhemoglobin resulted in less volume requirement and prolonged survival when compared to synthetic colloids (6% hetastarch) (34). In a rat model of LPS-induced shock, animals receiving an isotonic crystalloid and the carboxyhemoglobin compound showed improvement in renal cortical microcirculatory partial pressure in oxygen as well as intravital microscopy markers of improved skeletal muscle microcirculation, when compared to those receiving the isotonic crystalloid alone. The addition of norepinephrine did not further enhance cortical oxygen delivery. While there was no difference across groups in biomarkers of renal injury, the carboxyhemoglobin did not improve immunohistological markers of renal injury (35).

PEGylated carboxyhemoglobin has been further coupled with polynitroxyl. This chemical modification leverages the benefits of carboxylation, as discussed above, while providing superoxide dismutase and catalase-like activities. This modification affords antioxidant properties. This molecule was recently investigated in a guinea pig model of combined traumatic brain injury and hemorrhagic shock, which are the most lethal injuries in military casualties (36). Administration of this hyperoncotic solution following controlled cortical impact and hemorrhagic shock improved perfusion and reduced neuronal injury compared to lactated Ringer's.

Hemopure® (or HBOC-201) is another promising HBOC. It is a glutaraldehyde-polymerized, bovine hemoglobin. This compound has been studied in large animals for selective aortic arch perfusion (SAAP), an advanced endovascular resuscitation technique combining aortic balloon occlusion and proximal perfusion. In combination with SAAP, this HBOC was associated with successful outcomes following traumatic cardiac arrest modeled in pigs (37, 38). Such approach will potentially allow for prompt, blood-free augmentation in arterial oxygen content following traumatic exsanguination in the prehospital settings.

Side effects associated with first-generation HBOC have in part been attributed to their small molecular size (39). Another product refinement effort, therefore, focuses on developing products made up of large molecular weight hemoglobin polymers. Larger HBOCs reduce extravasation and renal injury, as well as nitric oxide scavenging. Those benefits materialized in a rat study of hemorrhagic shock, where large polymerized bovine hemoglobin restored blood pressure and cardiac function similar to fresh whole blood. Stored whole blood, however, was associated with renal and hepatic injury (40). The effects of those products on coagulation have yet to be determined. For instance, Polyheme®, a now-discontinued product, enhanced fibrinolysis (41), which is already a concern in trauma patients. There is, therefore, a concern that hyperfibrinolysis might be a property shared across various HBOCs.

Another product under development leverages a heme-based protein found in the thermostable bacterium *Thermoanaerobacter tengcongensis*. This molecule has a higher affinity for oxygen than hemoglobin, which allows for preferential delivery to hypoxic tissue. It also has a lower affinity for nitric oxide, which theoretically will not present the same hypertensive complications observed with first-generation HBOC. Similar to other blood product substitutes, the molecule is polymerized and PEGylated to prolong half-life (42). Being smaller than a red blood cell, the compound travels more easily through the microcirculation. In an ovine model of myocardial injury, administration of Omniox® resulted in improved myocardial oxygenation and contractility compared to control (42).

Another compound derived from the lugworm (*Arenicola marina*) is also penetrating the market as a preservation solution for organ transplants. The molecule is a large hemoglobin molecule that spontaneously occurs in the polychaete. It can load 156 oxygen molecules for transport, which is much higher than the four molecules the mammalian hemoglobin carries (43). The molecule, when administered to rats subjected to controlled cortical impact, improved blood pressure and, importantly, improved brain tissue oxygenation transiently (44). If further studies continue to show similar success, this compound might provide a bridging tool for patients with traumatic brain injury in the prehospital setting.

Hemoglobin can also be encapsulated to delay removal from circulation (45). The encapsulation process protects the molecules from degradation and oxidation while allowing for oxygen movement across the membrane. Much remains to be done to evaluate the immunogenicity of individual constructs. However, chronic administration of one of these encapsulated compounds to rats did not lead to significant antibody formation or tissue injury (46). The hemoglobin molecule can also be encapsulated with other molecules that preserve its structure and function (carbonic anhydrase, antioxidant enzymes, methemoglobin reductase, 2,3 DPG) (47). One such example is the bioparticle Erythromer®. Human hemoglobin is encapsulated in a membrane with compounds to lower oxygen affinity, prevent oxidation and prolong half-life. The bioparticle remains smaller than a red blood cell, thereby allowing it to navigate more easily through the microcirculation. The product also has the potential to be freeze-dried. In the lung, due to the higher pH, a novel compound bound to the inner membrane of the construct simulates 2,3 DPG activity. When the pH decreases, the novel compound interacts with hemoglobin and facilitates oxygen release. This facilitated release promotes oxygen delivery to hypoxic tissues.

*In vitro* production of red blood cells has been another approach to overcoming short supplies in red blood cells (48, 49). Farmed red blood cells development will free blood banks from concerns arising from donor-induced contamination of blood products and would provide a sustainable source of red blood cells if scaled appropriately. The ideal farmed red blood cell should be universally compatible and lead to no immunologic reaction. Most research efforts in the field have been funded by the Defense Advanced Research Projects Agency and leverage human stem cells to grow cultured red blood cells.

Research focusing on non-hemoglobin-based oxygen transport has been a dynamic field owing to earlier complications associated with HBOC as well as intrinsic limitations of hemoglobin-derived substitutes. Of those, perfluorocarbons (PFC) have been heavily studied. PFCs are amphipathic molecules; they transport oxygen with a weaker bond than hemoglobin, which leads to a linear oxygen saturation curve rather than a sigmoidal curve. While this translates to the need for higher partial pressure in oxygen for similar oxygen loading, oxygen unloading to tissue is better. Once in emulsion, those molecules can circulate through the microcirculation more efficiently than red blood cells owing to their smaller size. Similar to many HBOCs, several PFCs compounds have failed to translate from bench to bedside, but research and development efforts are still ongoing. Recent hemorrhagic shock studies in swine failed to show a benefit of a newer molecule (dodecafluoropentane) co-administered with fresh frozen plasma when compared to whole blood (50).

Iron-containing porphyrins have also been studied but do not currently seem to be an active area of product development. A porphyrin molecule can be combined with an iron atom to replicate the heme molecule (51). This molecule has then been encapsulated into a PEGylated lipid bilayer (52).

### Novel Platelet Products

Platelets are certainly the most challenging blood product to collect, process, ship, and store. Furthermore, they are expensive, have a short shelf life (5–14 days), and carry a higher risk of transfusion complications than other blood products such as packed RBC and plasma (53). In human medicine, platelets comprise only 10% of blood transfusion yet results in 25% of the transfusion reactions (54). Platelet transfusions are occasionally needed in times of severe thrombocytopenia due to immune-mediated conditions, neoplasia, bone marrow suppression and disseminated intravascular coagulopathy (55). Additionally, in people, platelet transfusions have been recommended as part of a balanced hemostatic resuscitation strategy for individuals who have sustained severe trauma and blood loss (56). However, transfusion of canine platelets is uncommon in veterinary medicine due to the high cost, decreased availability, and short shelf life (typically 5–7 days at room temperature) of fresh platelets (57).

Artificial platelets will address some of these challenges. SynthoPlate® is made of liposomes that are “decorated” with multiple types of peptides on their surface. One peptide is a von Willebrand factor binding peptide, which facilitates adherence to circulating or tethered von Willebrand factor. Another peptide is a collagen-binding peptide allowing the liposome to adhere to exposed collagen. The third peptide is GP IIb-IIIa binding fibrinogen-mimetic peptide allowing the liposome to participate in aggregation with other platelets (58). SynthoPlate® is not designed to replicate all of the capabilities of natural platelets such as degranulation, contraction, and morphologic changes. However, SynthoPlate® can help active platelets adhere to surfaces as well as allow multiple platelets to bind to them, acting as an extender for the host platelets (59).

SynthoPlate® has shown promise in several animal studies. Early studies showed that a mixture of aggregation and adhesions peptides reduced tail vein bleeding time in mice compared to liposomes decorated with only one type of peptide or saline (59). Another interesting study examined the effect of SynthoPlate® in thrombocytopenic mice. In this study, the investigators showed that SynthoPlate® administered to thrombocytopenic mice reduced bleeding time in a dose-dependent fashion. Even more interesting, at the highest dose of SynthoPlate® administered, bleeding times in these mice returned to near control levels suggesting that SynthoPlate® can potentially control bleeding even in animals with very low levels of natural platelets (60). In a model of uncontrolled hemorrhage, mice were administered SynthoPlate® either before or after liver injury. Under both conditions, SynthoPlate® administration resulted in significantly decreased blood loss compared to animals that did not receive SynthoPlate® (61). Finally, a large animal model showed similar results. In this model, pigs with a femoral artery injury were administered a bolus of SynthoPlate® as part of a resuscitation strategy. Not only was bleeding reduced in the pigs administered SynthoPlate® but the authors also reported an improved survival. In the SynthoPlate® group there was 100% survival after 60 min and 75% survival after 90 min, while the control group showed a 25% survival after 60 min and 0% survival after 90 min (58).

Synthetic platelet products offer several benefits over natural and modified platelets. They are shelf stable at room temperature and are expected to have a robust shelf life. They can be produced in large quantities once manufacturing has been optimized. Cryopreserved and lyophilized platelet products need to be harvested from donors and then processed, which could affect the availability of the finished products. Additionally, all platelet products derived from donors carry some risk of transfusion reaction, although the incidence of transfusion reactions secondary to platelet administration in veterinary medicine is not well established (55, 57). Perhaps the most attractive aspect of these synthetic platelet products for veterinary professionals is that they are expected to have little antigenicity and have already proven to work in several species without significant immunologic reactions. This could provide veterinarians the ability to treat many species with platelet-like products that have not been available previously.

While this product is early in its development and studies have yet to be performed in small animals, there are numerous potential clinical indications for synthetic platelets in veterinary medicine particularly in resource-constrained practices. Synthetic platelets are not a substitute for live platelets; however, they can be made economically and are not immunogenic (as expected). They may provide a way for general practitioners and specialists to treat immune mediated thrombocytopenia, consumptive coagulopathies, and bone marrow suppression, among other conditions. Treating feline patients, where no licensed platelet products for this species exist and fresh feline platelet products are not routinely available, might be of particular interest. These synthetic platelets may be able to extend the effect of the remaining platelets in animals preventing a bleeding diathesis while clinicians treat the underlying disease.

Naturally occurring platelet-derived hemostatic agents have also been evaluated as a replacement for current platelet products. Extracellular vesicles are small (10–1,000 nm diameter; exosomes 50–100 nm; microvesicles 100–1,000 nm) particles that express similar surface proteins as the cells they are secreted from. They contain a broad range of mediators and are capable of interacting with other cells and transferring their content to a target-cell. Platelet-derived extracellular vesicles isolated from leukoreduced apheresis platelets had endothelium-sparing properties and stimulated whole blood aggregation. Those vesicles reduced blood loss in a mouse model of uncontrolled blood loss (62).

### Freeze-Dried/Lyophilized Platelets

The introduction of lyophilized platelets (LP) has been a promising development in platelet substitutes. Lyophilized canine platelets (StablePlate®) is an FDA-approved and commercially available product that may simplify and increase platelet administration to veterinary patients. These LPs are made by obtaining leukoreduced apheresis platelets from donor dogs. These platelets are stabilized with trehalose, a disaccharide found in organisms able to withstand intense dehydration, mixed with a buffering solution and lyophilized (63, 64). This process allows the LPs to be room stable for up to 12 months and can be rapidly reconstituted with sterile water. This product is licensed for use in thrombocytopenic dogs with uncontrolled bleeding. A similar human-derived product dubbed Thrombosomes® is currently being investigated for use in people (65). A similar product called Stasix has also been developed was used in a swine liver injury model. In this experiment, pigs underwent a liver injury and were treated with either a low dose of Stasix or saline. Eighty percentage of the pigs treated with Stasix survived while only 20% survived in the saline treated group. However, pathologic thrombi were noted in the Stasix treated group, suggesting there may be some safety concerns with this product (66).

In 2017, investigators reported on the safety of canine LPs in a coronary bypass canine model. The group performed coronary bypass on eight dogs and returned up to 33% of their circulating platelet count with LPs. The group reported normal coagulation parameters and no abnormal thrombus formation in these dogs (67). More recently, Goggs et al. performed a multicenter randomized clinical trial of LP vs. dimethyl sulfoxide stabilized cryopreserved canine platelets for severely thrombocytopenic dogs (68). The group found that at 1 h after infusion the LP group had lower bleeding scores than the cryopreserved platelet group, but there was no significant difference at 24 h between groups. Furthermore, there was no difference between groups in platelet count change, need for additional red blood cell transfusions or survival to discharge. Studies are ongoing investigating the utility of canine LP for use in hemorrhagic shock and trauma.

### Fresh-Frozen Plasma Replacement Products

The development of an FDA-approved freeze-dried plasma (FDP) has been a priority for the Department of Defense. Current candidates include single-donor or pooled products. Heterogeneity in plasma hemostatic properties between donors is compensated for by pooling, thereby homogenizing clinical efficacy. Plasma can be purified by leukoreduction, solvent/detergent treatment, or microfiltration to obtain a product void of cells or cell debris. It can then be freeze-dried-lyophilized through a combination of low temperature, low pressure and low moisture circulating air or it can be spray dried by aerosolizing it into a high-temperature chamber to remove the moisture (69). FDP has regained prominence for trauma resuscitation in people. Three different countries are now manufacturing FDP for people (70), and it has been used with great success on the battlefield during the recent conflicts (71, 72). Beyond its use on the battlefield, FDP has shown to be efficacious in use for civilian traumas. In a study of aeromedical evacuation civilian trauma patients in the United Kingdom, patients transfused with FDP prehospital had a decreased need for red cell transfusions and decreased time to administration of blood products (73). FDP offers numerous advantages over FFP. FDP is shelf-stable at room temperatures and can be quickly reconstituted in <5 min with sterile water. FDP is currently recommended by the Department of Defense to resuscitate individuals in hemorrhagic shock when whole blood is not available or as part of a balanced resuscitation strategy (1 pRBC: 1 plasma: 1 platelets).

Currently, two companies have produced canine FDP with the help of Department of Defense funding. Bodevet Inc. has produced a pooled plasma product dubbed StablePlas®, which is expected to be FDA cleared and commercially available soon. Another company, Mantel Technologies, is also producing a high-quality canine FDP product. Both products can be manufactured in a plasma bag, can be reconstituted in under 5 min with sterile water, and have a long shelf life at room temperature. In a recent study, both products were considered to have hemostatic properties equivalent to canine fresh frozen plasma and in general retained their hemostatic capacity for up to 14 days after reconstitution when refrigerated (74). DoD funded studies are ongoing evaluating the *in vivo* use of canine FDP in hemorrhagic shock and trauma.

### Resuscitation Adjuncts

Regardless of the qualities of a novel fluid, there is also a growing interest in non-fluid-based resuscitation adjuncts for patients with hemorrhagic shock. Resuscitative endovascular balloon occlusion (or REBOA) is an endovascular hemorrhage control tool whereby a balloon-tipped catheter is inserted in the aorta (75). The balloon can be inflated in various locations of the aorta depending on the source of bleeding. Aortic occlusion can be complete, partial (allowing limited flow, continuously, around the balloon), or intermittent (the balloon is completely inflated and completely deflated at fixed intervals or after a set hypotension threshold is reached). REBOA is a bridging therapy that allows rapid stabilization of the patient until definitive care. However, it creates a severe ischemia-reperfusion syndrome for which the fluids of the future might be important and especially relevant. Other similar resuscitation tools that will be improved with new generation fluids include SAAP and extracorporeal membrane oxygenation.

## Limitations

Reviewing the literature to discuss fluids of the future outlines that much remains to be done to have a product with proven benefits in veterinary patients and that also overcame regulatory evaluation to make it to market. Synthetic platelets are the most likely product to become available to veterinary researchers to validate their efficacy in various clinical scenarios since they are FDA approved. The gap between bench to bedside is a major limitation for several of those products as many will not prove efficacious in our patients. This is the main focus of translational research. Animal data, especially in dogs and pigs, is the basis for translation into veterinary and human clinical practice. Many of the observed benefits of the products reviewed here might disappear in clinical studies. Veterinarians, physicians, bioengineers should strive to collaborate to ultimately evaluate those products to ultimately improve patient outcome. Such success will likely be the result of partnership between military agencies, civilian researchers, and private companies.

## Conclusions

Fluids are unquestionably an important part of resuscitation from several states of shock. Clinical and laboratory research have outlined several limitations of currently available products, which motivates efforts to develop new-generation fluids that will hopefully overcome those limitations. The search for the ideal fluid is driven by military research owing to specific operational constraints. Those efforts are also paralleled and supported by civilian efforts. Altogether, those research and development initiatives are likely to benefit both civilian and military veterinary patients. There are several candidates covering a wide range of syndromes, many of those remain early in the spectrum of translation from laboratory research to clinical use.

## Author Contributions

GH and TE: manuscript drafting and editing. All authors contributed to the article and approved the submitted version.

## Conflict of Interest

GH is a stockholder of Certus Critical Care, which engages in REBOA catheter development. The remaining author declare that the research was conducted in the absence of any commercial or financial relationships that could be construed as a potential conflict of interest.

## References

[1] Eastridge BJ Hardin M Cantrell J Oetjen-Gerdes L Zubko T Mallak C Died of wounds on the battlefield: causation and implications for improving combat casualty care. J Trauma. (2011) 71(1 Suppl.):S4–8. 10.1097/TA.0b013e318221147b 21795876

[2] Holcomb JB McMullin NR Pearse L Caruso J Wade CE Oetjen-Gerdes L. Causes of death in U.S. Special Operations Forces in the global war on terrorism: 2001-2004. Ann Surg. (2007) 245:986–91. 10.1097/01.sla.0000259433.03754.98 17522526 PMC1876965

[3] Eastridge BJ Mabry RL Seguin P Cantrell J Tops T Uribe P. Death on the battlefield (2001-2011): implications for the future of combat casualty care. J Trauma Acute Care Surg. (2012) 73(6 Suppl. 5):S431–7. 10.1097/TA.0b013e3182755dcc 23192066

[4] Shackford SR Mackersie RC Holbrook TL Davis JW Hollingsworth-Fridlund P Hoyt DB. The epidemiology of traumatic death. A population-based analysis. Arch Surg Chic Ill. (1993) 128:571–5. 10.1001/archsurg.1993.01420170107016 8489391

[5] Edwards T Scott L Gonyeau K Howard E Parker J Hall K Comparison of trauma sustained by civilian dogs and deployed military working dogs. J Vet Emerg Crit Care. (2019) 29:S49 10.1111/vec.13064 34014602

[6] Cecconi M De Backer D Antonelli M Beale R Bakker J Hofer C. Consensus on circulatory shock and hemodynamic monitoring. Task force of the European Society of Intensive Care Medicine. Intensive Care Med. (2014) 40:1795–815. 10.1007/s00134-014-3525-z 25392034 PMC4239778

[7] Hotchkiss RS Karl IE. Reevaluation of the role of cellular hypoxia and bioenergetic failure in sepsis. JAMA. (1992) 267:1503–10. 10.1001/jama.267.11.1503 1538541

[8] Brealey D Brand M Hargreaves I Heales S Land J Smolenski R. Association between mitochondrial dysfunction and severity and outcome of septic shock. Lancet Lond Engl. (2002) 360:219–23. 10.1016/S0140-6736(02)09459-X 12133657

[9] Hess JR Brohi K Dutton RP Hauser CJ Holcomb JB Kluger Y The coagulopathy of trauma: a review of mechanisms. J Trauma. (2008) 65:748–54. 10.1097/TA.0b013e3181877a9c 18849786

[10] Oller L Dyer WB Santamaria L Largo C Javidroozi M Shander A. The effect of a novel intravenous fluid (Oxsealife®) on recovery from haemorrhagic shock in pigs. Assoc Anesth. (2019) 74:765–77. 10.1111/anae.14627 30920660

[11] Wickramaratne N Kenning K Reichstetter H Blocher C Li R Aboutanos M. Acute resuscitation with polyethylene glycol-20k: a thromboelastographic analysis. J Trauma Acute Care Surg. (2019) 87:322–30. 10.1097/TA.0000000000002332 31033892

[12] Khoraki J Wickramaratne N Kang HS Xu H Archambault C Blocher C. Superior survival outcomes of a polyethylene glycol-20k based resuscitation solution in a preclinical porcine model of lethal hemorrhagic shock. Ann Surg. (2020). 10.1097/SLA.0000000000004070. [Epub ahead of print]. 32773641

[13] Dobson GP Letson HL. Far forward gaps in hemorrhagic shock and prolonged field care: an update of ALM fluid therapy for field use. J Spec Oper Med. (2020) 20:128–34. 32969018 10.55460/06VT-9IH4

[14] Letson HL Dobson GP. Adenosine, lidocaine, and Mg2+ (ALM) resuscitation fluid protects against experimental traumatic brain injury. J Trauma Acute Care Surg. (2018) 84:908–16. 10.1097/TA.0000000000001874 29554045

[15] Granfeldt A Letson HL Dobson GP Shi W Vinten-Johansen J Tønnesen E Adenosine, lidocaine and Mg2+ improves cardiac and pulmonary function, induces reversible hypotension and exerts anti-inflammatory effects in an endotoxemic porcine model. Crit Care Lond Engl. (2014) 18:682 10.1186/s13054-014-0682-y PMC4301798 25497775

[16] Letson H Dobson G. Adenosine, lidocaine and Mg2+ (ALM) fluid therapy attenuates systemic inflammation, platelet dysfunction and coagulopathy after non-compressible truncal hemorrhage. PLoS ONE. (2017) 12:e0188144. 10.1371/journal.pone.0188144 29145467 PMC5690633

[17] Cotton BA Guy JS Morris JAJ Abumrad NN. The cellular, metabolic, and systemic consequences of aggressive fluid resuscitation strategies. Shock. (2006) 26:115–21. 10.1097/01.shk.0000209564.84822.f2 16878017

[18] Howard JT Kotwal RS Stern CA Janak JC Mazuchowski EL Butler FK. Use of combat casualty care data to assess the US military trauma system during the Afghanistan and Iraq Conflicts, 2001-2017. JAMA Surg. (2019) 154:600–8. 10.1001/jamasurg.2019.0151 30916730 PMC6583837

[19] Stefanetti V Miglio A Cappelli K Capomaccio S Sgariglia E Marenzoni ML. Detection of bacterial contamination and DNA quantification in stored blood units in 2 veterinary hospital blood banks. Vet Clin Pathol. (2016) 45:406–10. 10.1111/vcp.12372 27642138

[20] Miglio A Stefanetti V Antognoni MT Cappelli K Capomaccio S Coletti M. Stored canine whole blood units: what is the real risk of bacterial contamination? J Vet Intern Med. (2016) 30:1830–7. 10.1111/jvim.14593 27734567 PMC5115181

[21] Levy JH. Hemoglobin-based oxygen carriers for reversing hypotension and shock: “NO” way, “NO” how? Anesthesiology. (2011) 114:1016–8. 10.1097/ALN.0b013e318215e20b 21521965

[22] Jahr J. Do approved blood substitutes reduce myocardial infarction size: is this the critical question? Br J Anaesth. (2009) 103:470–1. 10.1093/bja/aep227 19749116

[23] Mackenzie CF Dubé GP Pitman A Zafirelis M. Users guide to pitfalls and lessons learned about HBOC-201 during clinical trials, expanded access, and clinical use in 1,701 patients. Shock. (2019) 52(1S Suppl. 1):92–9. 10.1097/SHK.0000000000001038 29076972

[24] Natanson C Kern SJ Lurie P Banks SM Wolfe SM Cell-free hemoglobin-based blood substitutes and risk of myocardial infarction and death: a meta-analysis. JAMA. (2008) 299:2304–12. 10.1001/jama.299.19.jrv80007 18443023 PMC10802128

[25] Weiskopf RB. Hemoglobin-based oxygen carriers: disclosed history and the way ahead: the relativity of safety. Anesth Analg. (2014) 119:758–60. 10.1213/ANE.0000000000000401 25232689

[26] Alayash AI. Setbacks in blood substitutes research and development: a biochemical perspective. Clin Lab Med. (2010) 30:381–9. 10.1016/j.cll.2010.02.009 20513557

[27] Alayash AI. Mechanisms of toxicity and modulation of hemoglobin-based oxygen carriers. Shock. (2019) 52(1S Suppl 1.):41–9. 10.1097/SHK.0000000000001044 29112106 PMC5934345

[28] Sen Gupta A. Bio-inspired nanomedicine strategies for artificial blood components. Wiley Interdiscip Rev Nanomed Nanobiotechnol. (2017) 9. 10.1002/wnan.1464 28296287 PMC5599317

[29] Silverman TA Weiskopf RB. Hemoglobin-based oxygen carriers: current status and future directions. Transfusion. (2009) 49:2495–515. 10.1111/j.1537-2995.2009.02356.x 19903298

[30] Fitzgerald MC Chan JY Ross AW Liew SM Butt WW Baguley D. A synthetic haemoglobin-based oxygen carrier and the reversal of cardiac hypoxia secondary to severe anaemia following trauma. Med J Aust. (2011) 194:471–3. 10.5694/j.1326-5377.2011.tb03064.x 21534906

[31] Powanda DD Chang TMS. Cross-linked polyhemoglobin-superoxide dismutase-catalase supplies oxygen without causing blood-brain barrier disruption or brain edema in a rat model of transient global brain ischemia-reperfusion. Artif Cells Blood Substit Immobil Biotechnol. (2002) 30:23–37. 10.1081/BIO-120002725 12000224

[32] Abuchowski A. SANGUINATE (PEGylated carboxyhemoglobin bovine): mechanism of action and clinical update. Artif Organs. (2017) 41:346–50. 10.1111/aor.12934 28397407

[33] Guerci P Ergin B Kapucu A Hilty MP Jubin R Belohlavek J. Effect of polyethylene-glycolated carboxyhemoglobin on renal microcirculation in a rat model of hemorrhagic shock. Anesthesiology. (2019) 131:1110–24. 10.1097/ALN.0000000000002932 31490291

[34] Nugent WH Sheppard FR Dubick MA Cestero RF Darlington DN Jubin R. Microvascular and systemic impact of resuscitation with PEGylated carboxyhemoglobin-based oxygen carrier or hetastarch in a rat model of transient hemorrhagic shock. Shock. (2020) 53:493–502. 10.1097/SHK.0000000000001370 31045989

[35] Guerci P Ergin B Kandil A Ince Y Heeman P Hilty MP. Resuscitation with PEGylated carboxyhemoglobin preserves renal cortical oxygenation and improves skeletal muscle microcirculatory flow during endotoxemia. Am J Physiol Renal Physiol. (2020) 318:F1271–83. 10.1152/ajprenal.00513.2019 32281418

[36] Seno S Wang J Cao S Saraswati M Park S Simoni J. Resuscitation with macromolecular superoxide dismutase/catalase mimetic polynitroxylated PEGylated hemoglobin offers neuroprotection in guinea pigs after traumatic brain injury combined with hemorrhage shock. BMC Neurosci. (2020) 21:22. 10.1186/s12868-020-00571-7 32404052 PMC7222507

[37] Manning JE Katz LM Pearce LB Batson DN McCurdy SL Gawryl MS. Selective aortic arch perfusion with hemoglobin-based oxygen carrier-201 for resuscitation from exsanguinating cardiac arrest in swine. Crit Care Med. (2001) 29:2067–74. 10.1097/00003246-200111000-00005 11700396

[38] Hoops HE Manning JE Graham TL McCully BH McCurdy SL Ross JD. Selective aortic arch perfusion with fresh whole blood or HBOC-201 reverses hemorrhage-induced traumatic cardiac arrest in a lethal model of noncompressible torso hemorrhage. J Trauma Acute Care Surg. (2019) 87:263–73. 10.1097/TA.0000000000002315 31348400

[39] Rice J Philbin N Light R Arnaud F Steinbach T McGwin G. The effects of decreasing low-molecular weight hemoglobin components of hemoglobin-based oxygen carriers in swine with hemorrhagic shock. J Trauma. (2008) 64:1240–57. 10.1097/TA.0b013e318058245e 18469646

[40] Williams AT Lucas A Muller CR Munoz C Bolden-Rush C Palmer AF. Resuscitation from hemorrhagic shock with fresh and stored blood and polymerized hemoglobin. Shock. (2020) 54:464–73. 10.1097/SHK.0000000000001530 32097242 PMC7442586

[41] Morton AP Moore EE Moore HB Gonzalez E Chapman MP Peltz E. Hemoglobin-based oxygen carriers promote systemic hyperfibrinolysis that is both dependent and independent of plasmin. J Surg Res. (2017) 213:166–70. 10.1016/j.jss.2015.04.077 28601310 PMC5467451

[42] Boehme J Le Moan N Kameny RJ Loucks A Johengen MJ Lesneski AL. Preservation of myocardial contractility during acute hypoxia with OMX-CV, a novel oxygen delivery biotherapeutic. PLoS Biol. (2018) 16:e2005924. 10.1371/journal.pbio.2005924 30335746 PMC6193608

[43] Marchand A Crepin N Roulland I Semence F Domergue V Zal F. Application of HBOCs electrophoretic method to detect a new blood substitute derived from the giant extracellular haemoglobin of lugworm. Drug Test Anal. (2017) 9:1762–7. 10.1002/dta.2127 27787946

[44] Moon-Massat P Mullah SH-E-R Abutarboush R Saha BK Pappas G Haque A. Cerebral vasoactivity and oxygenation with oxygen carrier M101 in rats. J Neurotrauma. (2017) 34:2812–22. 10.1089/neu.2015.3908 26161914

[45] Liu X Jansman MMT Hosta-Rigau L. Haemoglobin-loaded metal organic framework-based nanoparticles camouflaged with a red blood cell membrane as potential oxygen delivery systems. Biomater Sci. (2020) 8:5859–73. 10.1039/D0BM01118E 32930196

[46] Yadav VR Nag O Awasthi V. Biological evaluation of liposome-encapsulated hemoglobin surface-modified with a novel PEGylated nonphospholipid amphiphile. Artif Organs. (2014) 38:625–33. 10.1111/aor.12304 24749870 PMC4146621

[47] Sen Gupta A. Hemoglobin-based oxygen carriers: current state-of-the-art and novel molecules. Shock. (2019) 52(1S Suppl. 1):70–83. 10.1097/SHK.0000000000001009 31513123 PMC6874912

[48] Seghatchian J. The secrets of human stem cell-derived transfusable RBC for targeted large-scale production and clinical applications: a fresh look into what we need most and lessons to be learned. Transfus Apher Sci. (2020) 59:102862. 10.1016/j.transci.2020.102862 32620410 PMC7320703

[49] Christaki E-E Politou M Antonelou M Athanasopoulos A Simantirakis E Seghatchian J. Ex vivo generation of transfusable red blood cells from various stem cell sources: a concise revisit of where we are now. Transfus Apher Sci. (2019) 58:108–12. 10.1016/j.transci.2018.12.015 30616958

[50] Bonanno AM Graham TL Wilson LN Madtson BM Ross JD. Efficacy of the perfluorocarbon dodecafluoropentane as an adjunct to pre-hospital resuscitation. PLoS ONE. (2018) 13:e0207197. 10.1371/journal.pone.0207197 30496190 PMC6264877

[51] Kitagishi H Mao Q Kitamura N Kita T. HemoCD as a totally synthetic artificial oxygen carrier: improvements in the synthesis and O(2) /CO discrimination. Artif Organs. (2017) 41:372–80. 10.1111/aor.12870 28326558

[52] Kakizaki T Kobayashi K Komatsu T Nishide H Tsuchida E. Lipidheme-microsphere (LH-M). A new type of totally synthetic oxygen carrier and its oxygen carrying ability. Artif Cells Blood Substit Immobil Biotechnol. (1994) 22:933–8. 10.3109/10731199409117932 7994420

[53] Annen K Olson JE. Optimizing platelet transfusions. Curr Opin Hematol. (2015) 22:559–64. 10.1097/MOH.0000000000000188 26390163

[54] Garraud O Cognasse F Tissot J-D Chavarin P Laperche S Morel P. Improving platelet transfusion safety: biomedical and technical considerations. Blood Transfus Trasfus Sangue. (2016) 14:109–22. 10.2450/2015.0042-15 26674828 PMC4781777

[55] Hux BD Martin LG. Platelet transfusions: treatment options for hemorrhage secondary to thrombocytopenia. J Vet Emerg Crit Care San Antonio Tex 2001. (2012) 22:73–80. 10.1111/j.1476-4431.2011.00706.x 23016744

[56] Cannon JW Khan MA Raja AS Cohen MJ Como JJ Cotton BA. Damage control resuscitation in patients with severe traumatic hemorrhage: a practice management guideline from the Eastern Association for the Surgery of Trauma. J Trauma Acute Care Surg. (2017) 82:605–17. 10.1097/TA.0000000000001333 28225743

[57] Callan MB Appleman EH Sachais BS. Canine platelet transfusions. J Vet Emerg Crit Care. (2009) 19:401–15. 10.1111/j.1476-4431.2009.00454.x 19821881

[58] Hickman DA Pawlowski CL Shevitz A Luc NF Kim A Girish A. Intravenous synthetic platelet (SynthoPlate) nanoconstructs reduce bleeding and improve “golden hour” survival in a porcine model of traumatic arterial hemorrhage. Sci Rep. (2018) 8:3118. 10.1038/s41598-018-21384-z 29449604 PMC5814434

[59] Modery-Pawlowski CL Tian LL Ravikumar M Wong TL Sen Gupta A. *In vitro* and *in vivo* hemostatic capabilities of a functionally integrated platelet-mimetic liposomal nanoconstruct. Biomaterials. (2013) 34:3031–41. 10.1016/j.biomaterials.2012.12.045 23357371

[60] Shukla M Sekhon UDS Betapudi V Li W Hickman DA Pawlowski CL. *In vitro* characterization of SynthoPlate^TM^ (synthetic platelet) technology and its *in vivo* evaluation in severely thrombocytopenic mice. J Thromb Haemost. (2017) 15:375–87. 10.1111/jth.13579 27925685 PMC5305617

[61] Dyer MR Hickman D Luc N Haldeman S Loughran P Pawlowski C. Intravenous administration of synthetic platelets (SynthoPlate) in a mouse liver injury model of uncontrolled hemorrhage improves hemostasis. J Trauma Acute Care Surg. (2018) 84:917–23. 10.1097/TA.0000000000001893 29538234 PMC5970031

[62] Miyazawa B Trivedi A Togarrati PP Potter D Baimukanova G Vivona L. Regulation of endothelial cell permeability by platelet-derived extracellular vesicles. J Trauma Acute Care Surg. (2019) 86:931–42. 10.1097/TA.0000000000002230 31124890 PMC7381393

[63] Bynum JA Meledeo MA Peltier GC McIntosh CS Taylor AS Montgomery RK. Evaluation of a lyophilized platelet-derived hemostatic product. Transfusion. (2019) 59:1490–8. 10.1111/trf.15167 30980737

[64] Goggs R Cremer S Brooks MB. Evaluation of cytokine concentrations in a trehalose-stabilised lyophilised canine platelet product: a preliminary study. Vet Rec Open. (2020) 7:e000366. 10.1136/vetreco-2019-000366 32821395 PMC7418665

[65] Fitzpatrick MG Novel platelet products under development for the treatment of thrombocytopenia or acute hemorrhage. Sci Direct. (2019) 58:7–11. 10.1016/j.transci.2018.12.010 30718153

[66] Hawksworth JS Elster EA Fryer D Sheppard F Morthole V Krishnamurthy G. Evaluation of lyophilized platelets as an infusible hemostatic agent in experimental non-compressible hemorrhage in swine. J Thromb Haemost. (2009) 7:1663–71. 10.1111/j.1538-7836.2009.03562.x 19656278

[67] Todd MG Anne SH Arthur PB Mark DJ G Michael F Safety evaluation of lyophilized canine platelets in a model of coronary artery bypass graft (CABG). Transfusion. (2017) 57:21A.

[68] Goggs R Brainard BM LeVine DN Calabro J Harrell K Mills T. Lyophilized platelets versus cryopreserved platelets for management of bleeding in thrombocytopenic dogs: a multicenter randomized clinical trial. J Vet Intern Med. (2020) 34:2384–97. 10.1111/jvim.15922 33016527 PMC7694820

[69] Inaba K. Freeze-dried plasma. J Trauma. (2011) 70(5 Suppl.):S57–8. 10.1097/TA.0b013e31821a6057 21841576

[70] Pusateri AE Given MB Schreiber MA Spinella PC Pati S Kozar RA. Dried plasma: state of the science and recent developments. Transfusion. (2016) 56 (Suppl. 2):S128–39. 10.1111/trf.13580 27100749

[71] Martinaud C Ausset S Deshayes AV Cauet A Demazeau N Sailliol A. Use of freeze-dried plasma in French intensive care unit in Afghanistan. J Trauma. (2011) 71:1761–4; discussion 1764–5. 10.1097/TA.0b013e31822a8fd5 22182886

[72] Sailliol A Martinaud C Cap AP Civadier C Clavier B Deshayes A-V. The evolving role of lyophilized plasma in remote damage control resuscitation in the French Armed Forces Health Service. Transfusion. (2013) 53 (Suppl. 1):65S−71S. 10.1111/trf.12038 23301975

[73] Oakeshott JE Griggs JE Wareham GM Lyon RM. Feasibility of prehospital freeze-dried plasma administration in a UK Helicopter Emergency Medical Service. Eur J Emerg Med. (2019) 26:373–8. 10.1097/MEJ.0000000000000585 30531322

[74] Edwards TH Meledeo MA Peltier GC Ruiz DD Henderson AF Travieso S Effects of refrigerated storage on hemostatic stability of four canine plasma products. Am J Vet Res. (2020) 81:964–72. 10.2460/ajvr.81.12.964 33251844

[75] Hoareau GL Tibbits EM Beyer CA Simon MA DeSoucy ES Faulconer ER. Resuscitative endovascular balloon occlusion of the aorta: review of the literature and applications to veterinary emergency and critical care. Front Vet Sci. (2019) 6:197. 10.3389/fvets.2019.00197 31275952 PMC6594359

